# The relationship between the monocyte-to-lymphocyte ratio and osteoporosis in postmenopausal females with T2DM: A retrospective study in Chinese population

**DOI:** 10.3389/fendo.2023.1112534

**Published:** 2023-02-20

**Authors:** Hailin Li, Xinqi Zhang, Qi Zhang, Qiong Zhang, Xueying Zhu, Tuxiu Xie, Shuo Wang

**Affiliations:** ^1^ Department of General Family Medicine, Renmin Hospital of Wuhan University, Wuhan, China; ^2^ Department of Endocrinology & Metabolism, Renmin Hospital of Wuhan University, Wuhan, China

**Keywords:** osteoporosis, T2DM, postmenopausal females, monocyte-to-lymphocyte, retrospective study

## Abstract

**Introduction:**

The risk of fragility fractures is increased in patients with type 2 diabetes mellitus (T2DM). Many reports indicate that inflammatory and immune responses are associated with osteoporosis and osteopenia. The monocyte-to-lymphocyte ratio (MLR) is a novel potential marker of inflammatory and immune responses. The present study evaluated the associations between the MLR and osteoporosis in postmenopausal females with T2DM.

**Methods:**

Data were obtained from 281 T2MD postmenopausal females, and divided into three groups: Osteoporosis group, osteopenia group and normal BMD group.

**Result:**

Data analyses revealed that the MLR was significantly lower in T2MD postmenopausal females with osteoporosis than in those with osteopenia and normal BMD. Logistic regression showed that the MLR was an independent protective factor for osteoporosis in postmenopausal females with T2DM (odds ratio [OR]: 0.015, 95% confidence interval [CI]: 0.000–0.772). Based on the receiver operating characteristic (ROC) curve, the MLR for diagnosing osteoporosis in postmenopausal females with T2DM was projected to be 0.1019, an area under the curve of 0.761 (95% CI: 0.685–0.838), a sensitivity of 74.8% and a specificity of 25.9%.

**Conclusions:**

The MLR have a high efficacy in diagnosis for osteoporosis in postmenopausal females with T2DM. MLR have the potential to be used as diagnosis marker for osteoporosis in postmenopausal females with T2DM.

## 1 Introduction

Fractures are a common complication of diabetes mellitus (DM) (1–3), and the risk of hip fracture is increased in patients with type 2 diabetes mellitus (T2DM) (4, 5), particularly in patients with T2DM and poorly controlled blood glucose (6). Research has also shown that patients with type 1 diabetes mellitus (T1DM) have decreased bone mineral density (BMD), and there is an increased risk of fracture in patients with T2DM (7, 8). T2DM is often associated with increased levels of pro-inflammatory cytokines, which have been implicated in the development of T2DM and microvascular and macrovascular complications. Chronic subclinical inflammation plays an important role in insulin resistance, metabolic syndrome and atherosclerosis (9, 10). Peripheral blood monocytes are classic indicators of inflammation, and peripheral blood monocytes are directly involved in osteoclast genesis. The monocyte-to-lymphocyte ratio (MLR) has been identified as a potential marker of inflammation in various conditions (11–13). Yue et al. previously reported that the MLR was an independent risk factor for diabetic retinopathy (14). The mechanism by which T2DM affects osteoporosis in postmenopausal females is unclear. The associations of the MLR and osteoporosis in postmenopausal females with T2DM have not been investigated to date. Therefore, the present study investigated the relationship between the MLR and osteoporosis in postmenopausal females with T2DM to evaluate the related factors influencing osteoporosis in postmenopausal females with T2DM.

## 2 Patients and methods

### 2.1 Study population

The patients were evaluated in the Renmin Hospital of Wuhan University. We assessed a total of 281 postmenopausal patients diagnosed with diabetes. Patients were excluded if they had type 1 diabetes mellitus, any acute inflammation, active infection, cancer, chronic liver diseases, or any diabetic complications except for osteopenia or osteoporosis. This research was approved by the ethics committee, and written informed consent was obtained from all enrolled patients.

### 2.2 Clinical examination and biochemical analysis

All subjects underwent dual energy X-ray bone densitometry to assess the *T*-score of BMD (DEXA, MEDILINK, France). The participants’ systolic blood pressures (SBP) and diastolic blood pressures (DBP) were measured after a 5-min rest using a sphygmomanometer. Venous blood samples were drawn after an overnight fast. All biochemical analyses were performed in our hospital, including a routine blood test, fasting blood glucose (FPG), fasting insulin (FINS), fasting C-peptide (FC-p), osteocalcin (OC), parathyroid hormone (PTH), serum uric acid (UA), 25-OH vitamin D3 (VitD3), total cholesterol (TC), triglyceride (TG), low-density lipoprotein cholesterol (LDL-C), C-terminal telopeptide of type I collagen (β-CTX), high-density lipoprotein cholesterol (HDL-C), and glycosylated hemoglobin A1c (HbA1c), The MLR was calculated as the ratio of monocytes to lymphocytes.

### 2.3 Statistical analysis

Non-parametric analyses were used to compare non-normally distributed numerical variables, and the results were expressed as the median and interquartile range. Logistic regression was used to analyze Osteoporosis in postmenopausal females with T2DM with risk factors. Receiver operating characteristic (ROC) curve analysis was used to compare the prognostic power of the MLR for Osteoporosis in postmenopausal females with T2DM. The predictive validities were quantified as areas under the ROC curves. The positive predictive value (95% confidence interval (CI)) and negative predictive value (95% CI) were calculated using MedCalc ver. 15.2.2. Other analyses were performed using SPSS 22.0, and p<0.05 was considered statistically significant.

## 3 Results

**Figure 1:**
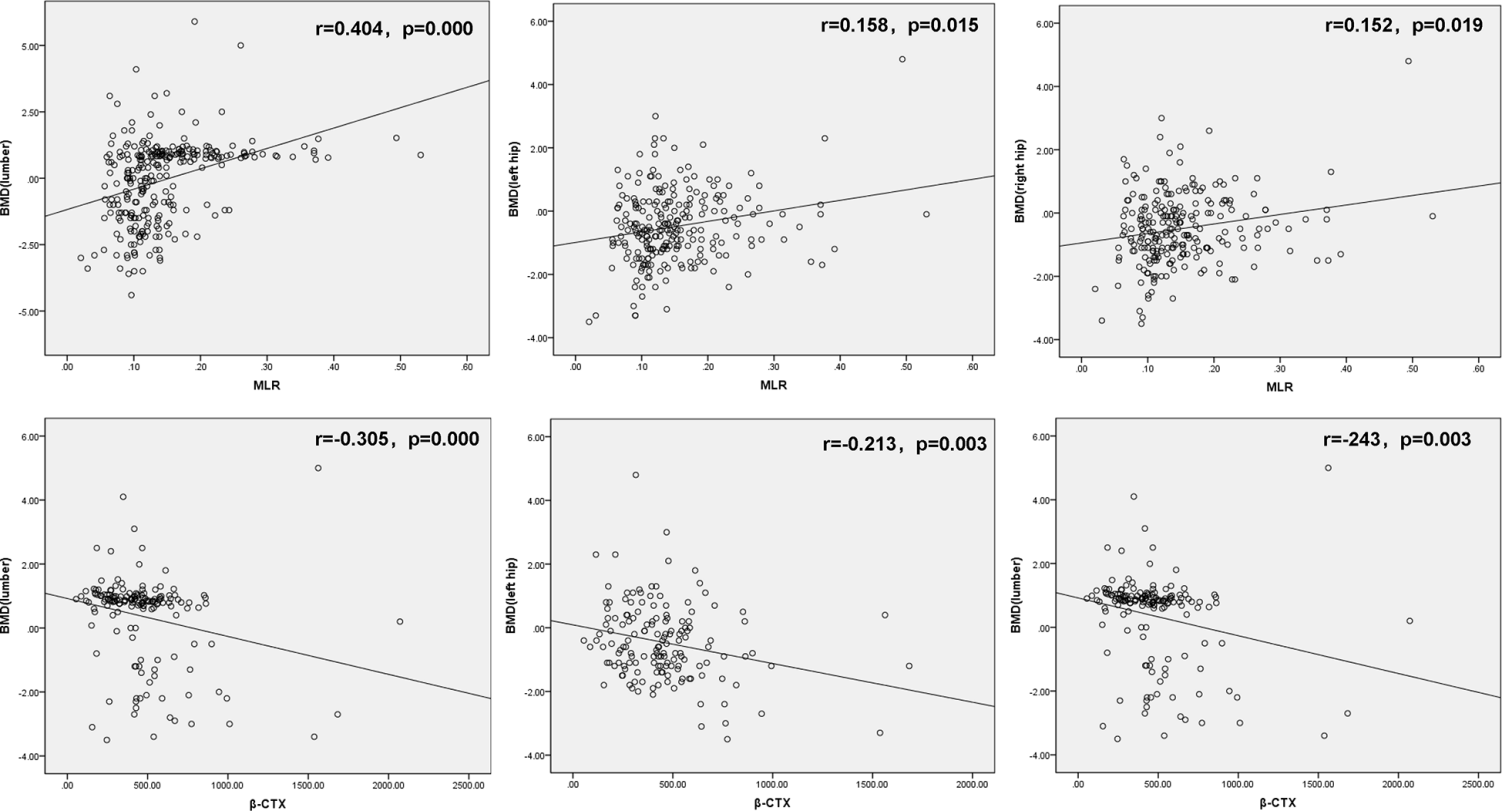
Correlation analysis of MLR with hip and lumbar BMD (up); Correlation analysis of MLR with hip and lumbar BMD (down). MLR, monocyte-to-lymphocyte ratio, β-CTX, C-terminal telopeptide of type I collagen and BMD, bone mineral density.

**Figure 2:**
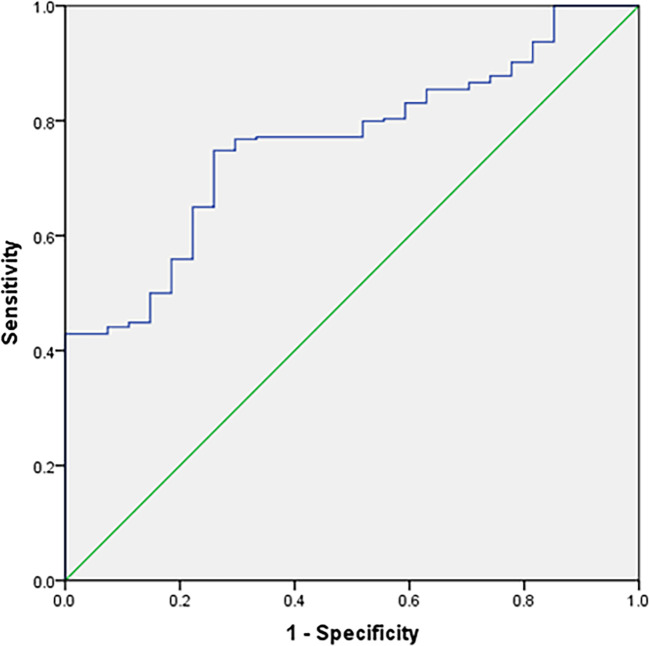
Receiver–operating characteristic (ROC) curve analysis for the monocyte-to-lymphocyte ratio as a predictor of Osteoporosis in postmenopausal females with T2DM (The sensitivity and specificity were 74.8% and 25.9%, respectively).

**Figure 3:**
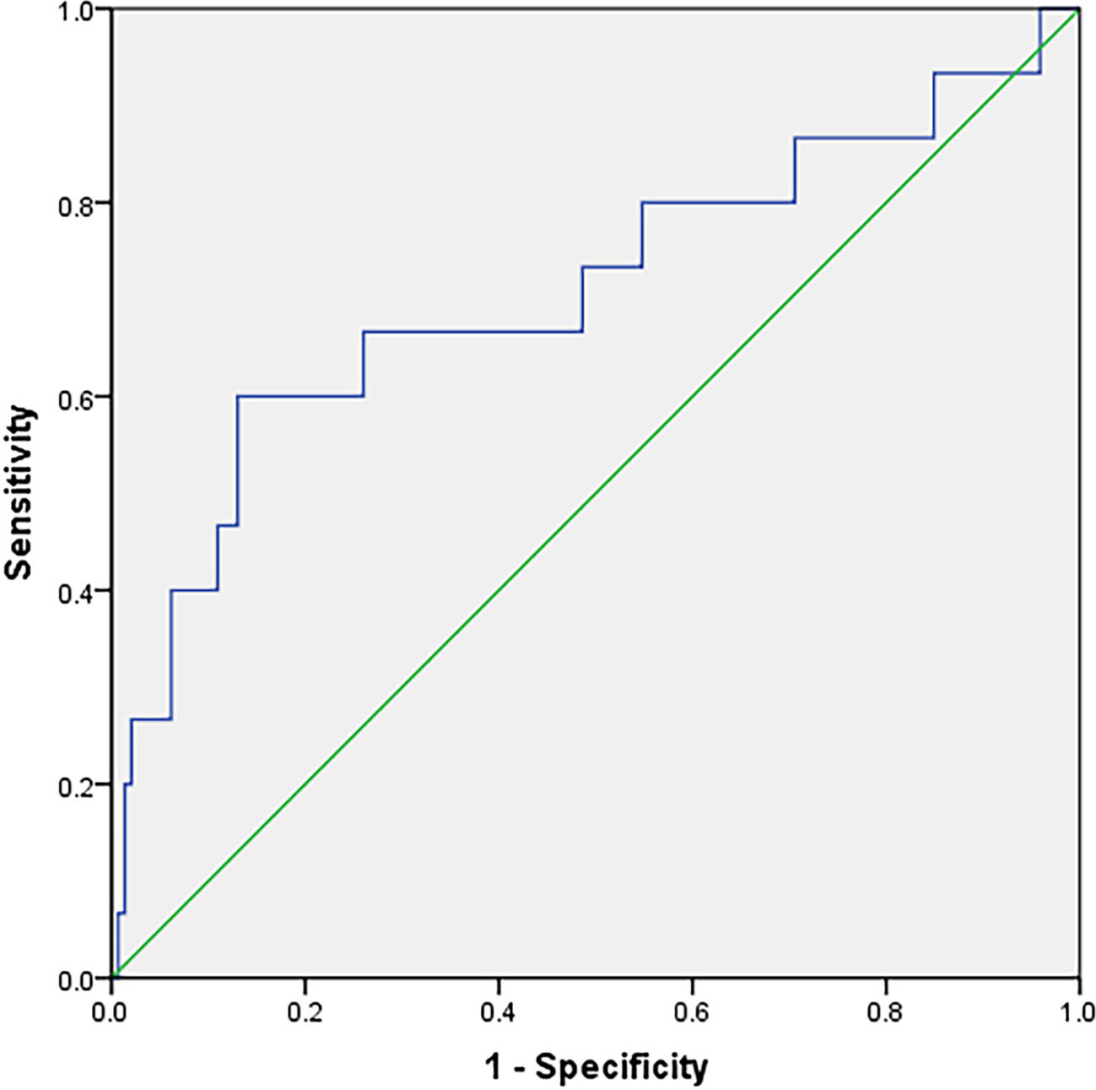
Receiver–operating characteristic (ROC) curve analysis for the β-CTX as a predictor of osteoporosis in postmenopausal females with T2DM (The sensitivity and specificity were 60.0% and 13.0%, respectively).

The postmenopausal females with T2DM were divided into three groups: Osteoporosis group, osteopenia group and normal BMD group. The clinical characteristics of the study population are shown in **Table 1**. The MLR were significantly lower in the osteoporosis group than those in the osteopenia group and the BMD normal group (**Table 1**). However, there was no significant difference in MLR between the osteopenia group and the normal BMD group. Age, menopausal years, β-CXT and OC were significantly higher in the osteoporosis group than those in the osteopenia group and the normal BMD group (**Table 1**). And the β-CTX in the osteopenia group was higher than that in the normal BMD group, but there was no statistical difference (p=0.087). Correlation analysis showed that MLR was positively correlated with hip and lumbar BMD, and β-CTX was negatively correlated with hip and lumbar BMD (**Figure 1**). Logistic regression analysis showed that the MLR was an independent protective factor for Osteoporosis in postmenopausal females with T2DM, while age and menopausal years were independent risk factor for Osteoporosis in postmenopausal females with T2DM. However, β-CTX was not an independent risk factor for Osteoporosis in postmenopausal females with T2DM (**Table 2**). ROC analysis showed that MLR had high diagnosis efficacy T2DM with osteoporosis in postmenopausal, with a cut-off value of 0.1019. Additionally, the area under ROC (AUC) of the MLR was 0.761 (95% CI: 0.685–0.838), the sensitivity and specificity were 74.8% and 25.9% respectively (**Figure 2**). The diagnostic efficacy of β-CTX is inferior to that of MLR, with a cut-off value of 637.3, an area under the curve of 0.710 (95% CI: 0.544–0.875), a sensitivity of 60.0% and a specificity of 13.0% (**Figure 3**).

**Table 1:** Clinical characteristics in postmenopausal females with T2DM. vs.normal a, vs. Osteopenia b,.

Variables	Postmenopausal females with T2DM		
	Osteoporosis (n=27)	Osteopenia (n=115)	Normal (n=139)
Age (years)	65 (60-71) a	64 (58-70) a	58 (54-63)
Menopause(years)	20 (13-25) a,b	11 (6-18) a	4 (3-7)
DM duration (years)	13.0 (6.0-20.0) a,b	10.0 (4.0-14.0)	8.0 (3.8-14.0)
BMI (kg/m^2^)	23.4 (22.2-25.0) a,b	25.0 (23.0-27.0)	25.3 (23.0-28.3)
SBP (mmHg)	130.0 (120.0-152.0)	130.0 (120.0-150.0)	130.0 (120.0-145.0)
DBP (mmHg)	80.0 (70.0-88.0)	80.0 (75.0-84.0)	80.0 (71.8-90.0)
MLR	0.10 (0.88-0.11) a,b	0.13 (0.10-0.17)	0.13 (0.11-0.18)
TG (mmol/L)	1.20 (0.76-1.93) a,b	1.66 (1.17-2.67)	1.77 (1.15-2.73)
LDL-C (mmol/L)	3.16 (2.38-4.02)	3.22 (2.71-3.85)	3.30 (2.66-3.73)
HDL-C (mmol/L)	1.39 (1.08-1.58) a,b	1.16 (1.03-1.37)	1.11 (0.96-1.30)
UA (mmol/L)	273.0 (235.0-316.5)	277.0 (232.0-346.0)	280.0 (238.0-340.0)
TC (mmol/L)	5.04 (4.13-6.18)	5.01 (4.44-5.80)	5.17 (4.45-5.63)
FBG (mmol/L)	6.25 (5.55-8.52) a	7.58 (5.90-10.56)	7.77 (6.12-10.89)
FINS (mIU/L)	7.40 (5.99-11.07)	8.90 (6.88-14.86)	9.02 (6.82-13.56)
FC-p (pmol/L)	451.2 (293.5-581.3) a,b	542.3 (350.6-733.0)	564.0 (411.9-840.2)
HbA1C (%)	7.10 (6.38-8.95)	7.70 (6.70-9.68)	7.70 (6.60-9.70)
VitD3 (nmol/L)	8.91 (4.52-14.99)	9.46 (5.83-14.48)	11.58 (8.25-15.09)
β-CTX (pg/mL)	643.2 (416.9-994.4) a,b	451.0 (325.4-544.3)	400.8 (271.5-532.7)
OC (ng/mL)	25.74 (15.90-35.70) a,b	16.10 (13.25-20.33)	14.98 (10.73-19.39)
PTH (pg/mL)	42.04 (31.29-64.20)	36.64 (27.19-51.27)	33.57 (27.94-46.20)
Ca (mmol/L)	2.22 (2.14-2.33)	2.21 (2.15-2.28)	2.23(2.14-2.28)
P (mmol/L)	1.23 (1.14-1.39)	1.20 (1.07-1.32)	1.21 (1.10-1.29)
*T*-score of BMD			
lumbar spine (%)	-2.90 (-3.10–2.50) a,b	-1.00 (-1.60-0.74) a	0.90 (0.33-1.17) b
right hip (%)	-2.3 (-2.70–1.5) a,b	-1.10 (-1.43–0.76) a	0.1 (-0.3-0.75) b
left hip (%)	-2.2 (-3.1–1.2) a,b	-1.10 (-1.60-0.80) a	0.2 (-0.4-0.8) b

Data are expressed as the median (inter-quartile range) or percentage; BMI, body mass index, SBP: systolic blood pressure, DBP: diastolic blood pressure; MLR, monocyte-to-lymphocyte ratio, LDL-C, low-density lipoprotein cholesterol, TG, triglyceride, HDL-C, high-density lipoprotein cholesterol, UA, serum uric acid, TC, total cholesterol, FPG, fasting blood glucose, FINS, fasting insulin, FC-P, fasting C-peptide, HbA1C, glycosylated hemoglobin A1c, VitD3, 25-OH vitamin D3, β-CTX, C-terminal telopeptide of type I collagen, OC, osteocalcin, PTH, parathyroid hormone, Ca, Calcium, P: phosphorus, and BMD, bone mineral density.

**Table 2:** Logistic regression analysis showing independent predictors of Osteoporosis in postmenopausal females with T2DM.

Variables	OR	95% CI	p value
MLR	0.015	0.000-0.772	0.033
Age	0.426	0.235-0.774	0.005
Menopause	2.702	1.417-5.154	0.003
DM duration	1.307	0.855-1.998	0.216
FBG	0.390	0.209-1.843	0.620
HbA1C	0.809	0.242-2.705	0.731
OC	0.907	0.794-1.037	0.152
β-CTX	1.008	0.987-1.029	0.172

OR, odds ratio; 95% CI, 95% confidence interval; BMI, body mass index; MLR, monocyte-to-lymphocyte ratio; FPG, fasting blood glucose; HbA1C, glycosylated hemoglobin A1c; β-CTX, C-terminal telopeptide of type I collagen.

## 4 Discussion

The MLR has been studied in many subjects, this was the first time the MLR was used as a marker of the relationship between the MLR and osteoporosis in postmenopausal females with T2DM. Our results indicate that the MLR is significantly higher in non-osteoporosis than osteoporosis in postmenopausal females with T2DM, and the MLR was an independent protective factor for osteoporosis with T2DM in postmenopausal females.

T2DM is associated with impaired bones, it is possible that bone microvascular disease impairs the mechanotactic function of osteocytes, bone turnover and collagen properties (15). The cytokines TNF and IL-6 are increased in patients with obesity and metabolic syndrome, and in hyperglycemic states, tissues exposed to inflammatory cytokines, such as IL-1, IL-6 and TNF, produce reactive oxygen species (ROS) that directly affects the differentiation and survival of osteoclasts, osteoblasts and osteocytes (16). TNF also stimulates osteoclast genesis and inhibits osteoblast genesis (17). Counts of white blood cells and their subtypes are important inflammatory markers associated with the pathophysiologic process of many diseases. In addition, many findings have revealed the predictive effects of the platelet-to-lymphocyte ratio (PLR) and neutrophil-to-lymphocyte ratio (NLR), particularly in DM, acute coronary syndromes, and various cancers (18–20). The MLR is an important marker of inflammation and the immune response and is related to tumor prognosis (21) and the MLR is also an independent risk factor for diabetic retinopathy (14). Because monocytes and lymphocytes play a crucial role in the induction and maintenance of an immune response, the MLR is used as a maker to measure vaccine efficacy in malaria (11). RANK and CCR6 expressed on monocytes are targets for the regulation of bone resorption in rheumatoid arthritis and osteoporosis (22). Previously reported that under suitable stimulation conditions, monocytes/macrophages can differentiate into osteoclasts (23). Studies have also demonstrated that activated T cells can induce osteoclast genesis by up-regulating the production of inflammatory cytokines (24, 25). Th17 cells are a critical modulator of the pathogenesis of estrogen-deficient osteoporosis (26). Together, these studies indicate that the MLR may be correlated with antibody or T cell responses involved in osteoporosis and osteopenia. Moreover, inflammation and immune responses are involved in the pathological process of osteoporosis and osteopenia, the MLR may be useful as a marker of inflammation in these conditions.

β-CTX is commonly used in clinical evaluation of bone metabolism, but was found in our study using the MLR as a marker to predict osteoporosis in postmenopausal females with T2DM is more reliable than β-CTX. Both MLR and β-CTX showed significant differences in the osteoporosis group compared with the normal BMD group, but β-CTX in the osteopenia group was also significantly higher than that in the normal BMD group, while there was no difference in MLR between the osteopenia group and the normal BMD group, it is suggested that MLR has higher specificity for osteoporosis. The ROC curve also confirmed that the sensitivity and specificity of MLR as a diagnosis of osteoporosis were better than those of β-CTX, therefore MLR was a better predictor of osteoporosis in menopausal females with T2DM than β-CTX and the MLR can be easily measured and cheaper. Although our study showed that the MLR may be an independent protective factor for osteoporosis in postmenopausal females with T2DM, the mechanism for how the MLR impacts osteoporosis in postmenopausal females with T2DM is unclear and warrants further investigation. We also found that the osteoporosis group was older than the normal BMD group and that age was an independent risk factor for osteoporosis in postmenopausal females with T2DM; and the duration of menopause was also significantly higher in the osteoporosis group than in the other two groups, this is consistent with previous research findings.

There are some limitations of our study. First, by restricting the clinical sample, our study did not include osteoporosis in males with T2DM. Second, the MLR was significantly higher in osteopenia group and normal BMD group, however, monocytes exhibit the potential to differentiate into osteoclasts. The increase of monocyte ratio leads to the increase of BMD, which needs further research to verify.

In conclusion, the MLR was significantly lower in T2DM postmenopausal females with osteoporosis than in those with osteopenia and normal BMD. And the MLR was an independent protective factor for osteoporosis in postmenopausal females with T2DM.

Additionally, the MLR have a high efficacy in diagnosis for osteoporosis in postmenopausal females with T2DM. MLR have the potential to be used as diagnosis marker for osteoporosis in postmenopausal females with T2DM.

## Data availability statement

The original contributions presented in the study are included in the article/supplementary material. Further inquiries can be directed to the corresponding author.

## Ethics statement

The studies involving human participants were reviewed and approved by Clinical Ethics Committee, Renmin Hospital of Wuhan University. The patients/participants provided their written informed consent to participate in this study.

## Author contributions

HL and SW participated in the study design, figures, data interpretation and writing; XiZ, QioZ, QiZ, XuZ. and TX participated in data analysis, data interpretation and writing; HL participated in figures, SW participated in the literature search, figures, study design, data analysis, interpretation and writing. All authors contributed to the article and approved the submitted version.
